# Humanized FcεRI Expressed on Mouse Eosinophils Mediates IgE-Facilitated Eosinophil Antigen Presentation

**DOI:** 10.3390/cells14040301

**Published:** 2025-02-18

**Authors:** Haibin Wang, Jean-Pierre Kinet, Peter F. Weller

**Affiliations:** 1 Division of Allergy and Inflammation, Department of Medicine, Beth Israel Deaconess Medical Center, Harvard Medical School, 330 Brookline Avenue, CLS 943, Boston, MA 02215, USA; 2 Department of Pathology, Beth Israel Deaconess Medical Center, Harvard Medical School, 330 Brookline Avenue, CLS 943, Boston, MA 02215, USA

**Keywords:** eosinophils, antigen-presentation, IgE receptor, IgE, FcεRI

## Abstract

High-affinity IgE receptors (FcεRI) are expressed on human blood eosinophils and may be upregulated on eosinophils at sites of allergic inflammation including atopic dermatitis and allergic asthma. FcεRI engagement, however, fails to elicit “effector” responses from eosinophils. Thus, a functional role for FcεRI on eosinophils has been uncertain. We evaluated the role of FcεRI in enhancing eosinophil antigen presentation in vivo by using humanized FcεRI α chain (hFcεRIα) transgenic mice. Eosinophils from hFcεRIα transgenic mice expressed humanized FcεRIα, with higher levels of eosinophils from the bronchoalveolar lavage of experimental asthma than those from polymyxin-elicited peritoneal lavage. The hFcεRIα-bearing eosinophils instilled intratracheally (i.t.) into recipient wild-type mice migrated from airways into paratracheal lymph nodes (pLNs) and spleens. Eosinophils, pretreated in vitro with nitrophenyl-ovalbumin ((NP)-OVA) and anti-NP human IgE complexes and instilled i.t., presented NP antigen via hFcεRIα to T cells more effectively than those pretreated with NP-OVA only, as assessed by pLN cell proliferation. IgE/FcεRIα-facilitated eosinophil antigen presentation resulted in increased IL-4 but not INF-γ production by pLN cells, with a bias towards Th2 cytokine production. Furthermore, cross-linking hFcεRIα on eosinophils increased eosinophil expressions of T cell costimulatory proteins CD40, CD80, and CD86. Humanized FcεRIα on murine eosinophils functions to enhance eosinophil antigen presentation capacities by mediating IgE-facilitated antigen presentation and upregulating expression of requisite T cell costimulatory proteins. Thus, a functional, non-“effector” role for FcεRI on eosinophils is revealed through identifying a means by which IgE may act on eosinophils to mediate their immunomodulatory, enhanced antigen presentation capabilities.

## 1. Introduction

Eosinophilia and heightened IgE responses are frequent concomitant hallmarks of Th2-type responses in varied allergic diseases, including atopic dermatitis and asthma, and in helminth infections. Cell receptors for IgE include the low-affinity CD23 IgE receptor and the high-affinity IgE receptor, FcεRI [1]. Human eosinophils may express both IgE receptors, but activation of eosinophils via their IgE receptors has failed to elicit potential eosinophil cellular “effector” responses. Notably, IgE-mediated stimulation of human eosinophils has not: (1) elicited secretion of eosinophil granule cationic proteins, (2) enhanced leukotriene C_4_ generation, or (3) stimulated superoxide formation [2]. Potential functional interactions between IgE and eosinophils mediated via eosinophil-expressed FcεRI are undefined and have been fraught with uncertainties [3,4].

FcεRI expression in mice is restricted to mast cells and basophils, whereas in humans FcεRI is expressed on monocytes, Langerhans cells, dendritic cells, and eosinophils [5,6]. FcεRI is a tetramer (αβγ_2_) on effector cells (mast cells and basophils) and a trimer (αγ_2_) on antigen-presenting cells (APCs), including eosinophils, Langerhans cells, and dendritic cells) [5,7]. IgE is a unique antibody (Ab) that may upregulate expression of FcεRI on mast cells and basophils. The β chain promotes the maturation of α chain and its intracellular trafficking to the cell surface and thus increases FcεRI expression on basophils and mast cells. This augmented expression of FcεRI is attributable to increased cell surface β chain stabilization of α chain–IgE complexes that draw on intracellular stores of preformed endocytosed α chain not requiring de novo increases in mRNA transcription or increased α chain synthesis [8]. Cells (APCs) that lack a β chain but share a γ chain dimer have a variable range of FcεRI expression. Studies have only rarely reported positive correlations between FcεRI expression on human blood eosinophils and serum IgE titers [6]. IgE binds only to the α chain of FcεRI on either effector cells or APCs.

Eosinophils can function as both “effector” cells and APCs. We previously demonstrated that mouse airway eosinophils exposed to aerosolized antigen (Ag) migrated from the airway lumen to elicit Ag-specific and CD80- and CD86-dependent CD4^+^ T cell responses in paratracheal lymph nodes (pLNs) of previously Ag-sensitized mice [9]. We further demonstrated that Ag-loaded airway eosinophils functioned as “professional” APCs to prime naïve CD4^+^ T cells and to elicit an IL-4 dominant Th2 cytokine response [10]. Eosinophil functions as APCs have been confirmed by studies in mice and humans [11,12,13].

A function of FcεRI-bearing APCs is the specific uptake and processing of IgE-bound Ags, which is followed by T cell stimulation [5]. Although FcεRI expression may be minimal on peripheral blood eosinophils, it can be up-regulated on eosinophils at sites of allergic inflammation, including atopic dermatitis [14,15,16], allergen-induced rhinitis [17], and allergic asthma [18]. As 85–95% of FcεRIα-positive cells were eosinophils in the bronchoalveolar lavage (BAL) from atopic asthmatics [18], we hypothesized that targeting allergens to FcεRI on airway eosinophils by allergen-specific IgE, which is present within airway secretions together with inhaled allergens, would enhance eosinophil APC function. To test the hypothesis, we used humanized FcεRIα (hFcεRIα) transgenic mice [19]. Because of the lack of native FcεRI expression on mouse eosinophils [20], mice expressing humanized FcεRI provide a useful model of responses mediated through this receptor. We reported that hFcεRI on murine eosinophils can enhance eosinophil Ag presentation capacities by mediating IgE-facilitated Ag presentation and increasing eosinophil expression of requisite T cell costimulatory proteins. The findings provide novel insights into the functionality of FcεRI on eosinophils distinct from any roles of FcεRI in eosinophil effector functions.

## 2. Materials and Methods

### 2.1. Mice

Human FcεRIα (hFcεRIα) transgenic BALB/c mice, in which the murine α chain was deleted and the human FcεRI α chain was expressed under control of the human FcεRIα promoter [19], were provided by Prof. Jean-Pierre Kinet (Beth Israel Deaconess Medical Center, Harvard Medical School). Wild-type (WT) BALB/c mice were purchased from Charles River Laboratories (Wilmington, MA, USA). Mice were 8–12 weeks old and were maintained in our animal facility under protocols approved by the Institutional Animal Care and Use Committee of Beth Israel Deaconess Medical Center (Boston, MA, USA).

### 2.2. Preparation of Eosinophils

Eosinophils were recovered from peritoneal cavities of hFcεRIα transgenic mice after intraperitoneal (i.p.) injections of polymyxin, 200 μg in PBS, twice weekly for 8–10 weeks [21]. In many experiments, eosinophils were isolated from BAL of OVA-sensitized and aerosol-challenged mice. Eosinophils were purified by discontinuous Percoll density gradients followed by immunomagnetic beads as described [9]. In brief, four densities (1.085, 1.080, 1.075, and 1.070 gm/mL) of Percoll were prepared with PBS. Five milliliters of each decreasing density were sequentially layered in 50 mL plastic tubes, and cells in 5 mL PBS/serum were layered on top. After centrifugation (25 min, 1500× *g*, room temperature), eosinophils were between the 1.075 and 1.070 g/mL layers, with most macrophages on top of the gradients. Collected eosinophils were washed in PBS/serum and cultured for 1 h on plastic Petri dishes in RPMI-1640 supplemented with penicillin (100 U/mL), streptomycin (100 μg/mL), L-glutamine (2 mM), HEPES (10 mM), and 5% mouse serum to eliminate remaining adherent macrophages. Eosinophils were further purified by negative immunomagnetic selection (MACS; Miltenyi Biotec, Auburn, CA, USA) with anti-CD90- and anti-CD19-coated micromagnetic beads to remove contaminating lymphocytes. Purity of eosinophils was >99% as assessed by Diff Quick staining of cytospins, and viability of eosinophils was >99%.

### 2.3. Flow Cytometric Analysis of Eosinophil Surface hFcεRIα Expression

A monoclonal Ab (mAb) (15.1) that binds the IgE-binding domain of the α chain of the human FcεRI was used for detection and blocking studies [22]. Eosinophils from either peritoneal cavities or BAL of hFcεRIα mice were stained at 4 °C for 30 min with FITC-conjugated anti-hFcεRIα mAb (15.1) [22], provided by Jean-Pierre Kinet, or matched isotype IgG1 (MCA1209F, Serotec, Oxford, UK), and then fixed in 0.5% paraformaldehyde. Flow cytometry was performed with a FACScan (Becton Dickinson Immunocytometry Systems, San Jose, CA, USA).

### 2.4. In Vivo Migration of hFcεRIα-Bearing Eosinophils

Peritoneal eosinophils recovered from hFcεRIα mice were stained ex vivo with the red fluorescent dye, DiIC_16_(3) (Molecular Probes, Eugene, OR, USA), as described [10]. The DiIC_16_(3)-labeled eosinophils (1 × 10^6^ in 50 µL PBS) were intratracheally (i.t.) injected into recipient BALB/c mice. At indicated times after injection, pLNs and spleens were harvested, teased into single cell suspensions via a 40 μm filter in FACS buffer (2% FCS, PBS), and stained with FITC-anti-hFcεRI α mAb (15.1). Doubly fluorescent events were evaluated by flow cytometry, and numbers of double positive donor eosinophils within tissues were calculated by multiplying total tissue cells by corresponding percentage of donor eosinophils determined by two-color flow cytometry. DiIC_16_(3)-labeled eosinophils (10^6^ in 50 µL PBS) were also injected intravenously into tail veins of BALB/c mice and likewise assessed for their tissue dispositions.

### 2.5. Texas Red-OVA Internalization by Airway Eosinophils

Human FcεRIα and WT mice were sensitized intraperitoneally (i.p.) three times with 10 µg of OVA plus 1 mg Al(OH)_3_ in 0.2 mL PBS on days 1, 7, and 14. On days 21, 22, and 23, sensitized mice underwent inhalational challenges with OVA by daily exposures to aerosolized OVA (5% in PBS) delivered for 30 min by a DeVilbiss 646 nebulizer (DeVilbiss, Somerset, PA, USA). After the last aerosol challenge, 1.5 mg of Texas Red-OVA (Molecular Probes, Eugene, OR, USA) in 50 µL PBS was i.t. injected into the mice. Control mice, likewise OVA-sensitized and aerosol-challenged, received non-labeled OVA i.t. Six hours after injection, the mice were sacrificed and BAL was collected for isolation of eosinophils. Fluorescently positive (Texas Red-OVA^+^) eosinophils were identified by flow cytometry and microscopy.

### 2.6. Paratracheal Lymph Node Cell Proliferation Assay and Cytokine Release

Studies utilized a chimeric human IgE (cIgE) anti-4-hydroxy-3-nitro-phenacetyl (NP) Ab bearing human IgE (JVV8/1, Serotec, Oxford, UK) [23]. Five × 10^5^ polymyxin-elicited peritoneal eosinophils from hFcεRI α chain mice were pre-incubated for 30 min at 37 °C with NP-OVA only or with NP-OVA plus cIgE anti-NP (10 µg/mL) complexes at OVA concentrations of 100 µg/mL. Cells, washed in PBS, were injected into the tracheas of NP-OVA sensitized mice, which had been i.p. immunized with 10 μg of NP-OVA plus 1 mg Al(OH)_3_ 2 weeks earlier. Control mice received i.t. instillation of the same volume of 50 µL PBS, or eosinophils pre-treated with OVA only, NP-OVA only, or control human IgE (10 µg/mL) (clone# HE1, ThermoFisher, Waltham, MA, USA). To block hFcεRIα-mediated IgE-facilitated NP-OVA presentation, in some wells increasing concentrations of blocking anti-FcεRIα mAb (15.1) were added to incubations with eosinophils and NP-OVA-cIgE complexes. Three days after i.t. eosinophil transfer, pLNs were harvested and teased into single cell suspensions. Lymph node cells (3 × 10^5^) in triplicate were pulsed with 1 µCi of ^3^H-thymidine (NEN Life Science, Boston, MA, USA) per well and cultured for 16 h. Paratracheal LN proliferations were measured by liquid scintillation spectrometry. Data are represented as mean ± SD of triplicate wells, representative of 3 independent experiments. In addition, 3 days after i.t. eosinophil transfer, pLNs were also harvested, teased into single cells, and cultured for 16 h. Supernatants were collected for IL-4 and INF-γ measurements by ELISA, per optEIATM kits (PharMingen, San Diego, CA, USA). Data are presented as mean ± SD of triplicates, representative of 3 independent experiments.

### 2.7. FcεRI Cross-Linking and CD40, CD80 and CD86 Expression

Eosinophils purified from BAL of OVA-sensitized and -challenged hFcεRIα mice were incubated for 30 min with 10 µg/mL cIgE or 10 µg/mL anti-FcεRIα mAb (15.1) or isotype-matched control mIgG1 (Serotec, Oxford, UK) at 37 °C. After washing with culture medium, 10 µg/mL anti-hIgEFc (clone# B3102E8, Southern Biotech, Birmingham, AL, USA) or 10 µg/mL rat anti-mouse IgG1 (#04-6100, Invitrogen, Carlsbad, CA, USA) were added. Cells, collected after 18 h, were analyzed for CD40, CD80, and CD86 expression by flow cytometry and Western blotting. For flow cytometric analysis, eosinophils in PBS containing 1% BSA were incubated with FITC-conjugated anti-CD40, anti-CD80, anti-CD86, or FITC-isotypic IgG2a mAbs (Serotec, Oxford, UK) at final concentrations of 2 µg/mL at 4 °C for 30 min. After washing, cells were fixed in 0.5% paraformaldehyde and analyzed by flow cytometry (Becton Dickinson FACScan). For Western blotting, cells were lysed in PBS containing 1% NP-40 and protease inhibitors. Lysates were resolved by 6% SDS-PAGE and transferred onto polyvinylidene difluoride membranes. Blots were blocked overnight at 4 °C in Tris-buffered saline (TBS) containing 0.1% Tween 20 and 5% milk before incubations with goat anti-mouse CD40, CD80, or CD86 Abs (Serotec, Oxford, UK) diluted in TBS/0.1% Tween 20 for 2 h at room temperature. As loading controls, blots were probed for β-actin (rabbit anti-β-actin, Abcam #ab8227). After three washes in TBS/0.1% Tween 20, blots were incubated in HRP-conjugated anti-goat (ab97110, Abcam, Waltham, MA, USA) or anti-rabbit (Ab6721, Abcam, Waltham, MA, USA) secondary Ab at a 1:2500 dilution in TBS for 2 h. Blots were washed and developed by chemiluminescence (Pierce, Rockford, IL, USA).

## 3. Results

### 3.1. Expression of Humanized FcεRI on Mouse Eosinophils

**Figure 1:**
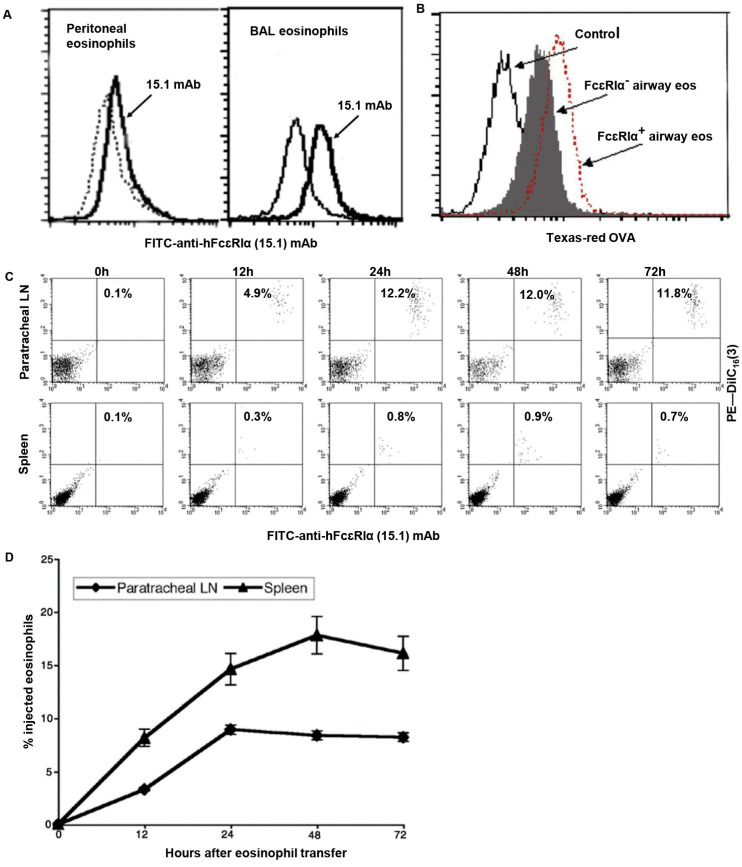
HFcεRIα expressing eosinophils take up Ag in the lungs and migrate to pLNs and spleens. (**A**) Eosinophils from peritoneal exudates or from BAL of OVA-sensitized and airways-challenged hFcεRIα mice expressed hFcεRI detected by anti-hFcεRI 15.1 mAb vs control IgG1 mAb. (**B**) In OVA-sensitized and airways-challenged mice, FcεRI-facilitated Texas Red-OVA Ag uptake by hFcεRIα eosinophils (red line) in comparison with WT eosinophils (filled) or eosinophils from control i.t. recipients of unlabeled OVA. (**C**) Eosinophils from hFcεRIα mice, stained with DiIC_16_(3) and instilled i.t into WT mice, migrated from the airways to pLNs and spleens. (**D**) Trafficking of DiIC_16_(3) and hFcεRIα double positive airways instilled eosinophils into pLNs and spleens, calculated by multiplying total tissue cells by percentage of DiIC_16_(3) and hFcεRIα double positive eosinophils vs numbers of instilled DiIC_16_(3) and hFcεRIα double positive eosinophils (SD, triplicates).

The expression of hFcεRIα on eosinophils from hFcεRIα mice was verified with anti-hFcεRI α chain specific mAb (15.1). By flow cytometry, both polymyxin-elicited peritoneal eosinophils and BAL eosinophils (Figure 1A) from OVA-sensitized and aerosol-challenged hFcεRIα mice expressed humanized FcεRI. No FcεRI was detected on eosinophils from control WT BALB/c mice (Figure S1A). Notably, BAL eosinophils from OVA-sensitized and -challenged hFcεRIα mice expressed significantly higher levels of hFcεRIα than elicited peritoneal eosinophils (Figure S1B). 

This resembled findings in humans that BAL eosinophils from patients with allergic asthma had elevated FcεRI expression compared with blood eosinophils [18].

### 3.2. FcεRI Facilitates Antigen Uptake by Airway Eosinophils in Vivo

We assessed whether the hFcεRIα receptor might function as an Ag-focusing mechanism and thereby facilitate allergen uptake by eosinophils in vivo. Both WT and hFcεRIα BALB/c mice were sensitized to OVA and subjected to three aerosol challenges with OVA. Texas Red-OVA was instilled i.t. after the last OVA aerosol challenge. Six h later, BAL eosinophils were purified and analyzed by flow cytometry. FcεRIα^+^ eosinophils from hFcεRIα mice took up more Texas Red-OVA than FcεRI^−^ eosinophils from WT mice (Figure 1B).

### 3.3. In Vivo Migration of hFcεRI-Bearing Eosinophils

For airway eosinophils to function as APCs, a requisite step would be the eosinophils’ capacity to migrate from the airway lumen into regional lymph nodes, where eosinophils might present Ag and activate Ag-specific T cells. To evaluate this trafficking capacity, we purified eosinophils from peritoneal exudates of hFcεRIα mice and labeled these eosinophils ex vivo with red fluorescent dye DiIC_16_(3). The red-labeled eosinophils were instilled i.t. into WT BALB/c recipient mice. By flow cytometry, donor eosinophils, identified as FITC mAb 15.1 and DiIC _16_(3) double positive cells, were detected in pLNs with a peak at 24 h after i.t. eosinophil transfer (Figure 1C), consonant with prior findings with DiIC _16_(3)-labeled eosinophils [9]. Moreover, FITC mAb 15.1 and DiIC _16_(3) double positive hFcεRIα eosinophils, instilled endobronchially, trafficked more distantly than pLNs into spleens with a peak 48 h after instillation (Figure 1C). Double positive hFcεRIα eosinophils, instilled endobronchially, were not detected in non-regional LNs, such as popliteal or peritoneal LNs. Twenty-four hours after i.t. injection, about 9% and 14% of the i.t instilled eosinophils had migrated into the pLN and spleen, respectively (Figure 1D). In control experiments, DiIC _16_(3)-labeled eosinophils, administered intravenously into WT BALB/c mice, trafficked to the spleen but not to pLNs or other LNs.

### 3.4. FcεRI-IgE-Allergen Complexes Facilitate Antigen Presentation In Vivo

**Figure 2:**
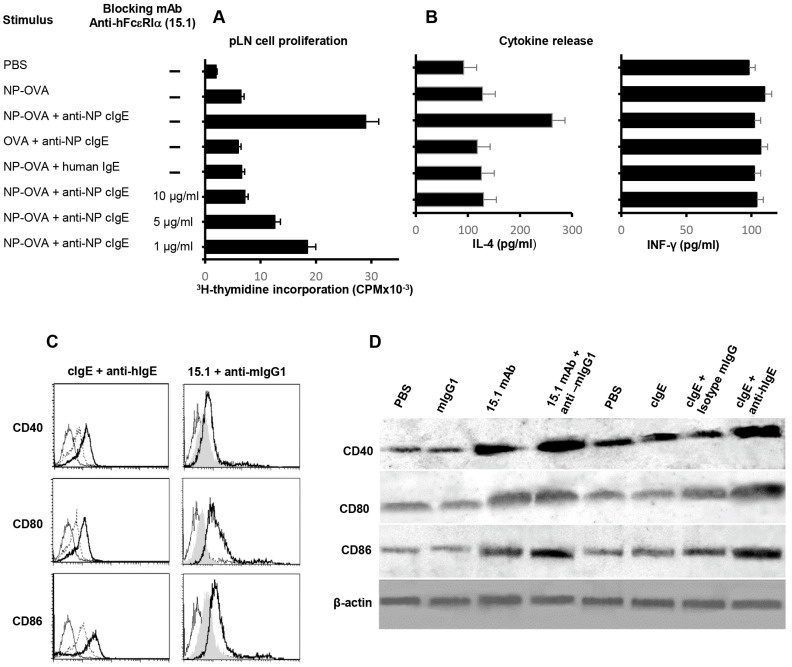
IgE-hFcεRI engagement facilitates eosinophil antigen presentation in vivo to enhance pLN T cell proliferation and IL-4 release and to increase eosinophil CD40, CD80, and CD86 expression. (**A**,**B**) hFcεRIα eosinophils, incubated with NP-OVA or NP-OVA and chimeric anti-NP human IgE, were injected i.t. into NP-OVA-immunized mice. Controls received i.t instillation of PBS or eosinophils treated with OVA, NP-OVA, or control human IgE. (**A**) To block hFcεRIα-mediated cIgE-facilitated NP-OVA presentation, anti-FcεRIα mAb (15.1) doses were used. Three days after eosinophil instillation, pLN cells were assayed for T cell proliferation (**B**) Three days after i.t. eosinophil transfer, pLN cells were cultured and assayed for IL-4 and INF-γ release. (**C**) FcεRI engagement increases expressions of CD40, CD80, and CD86 on airway eosinophils. On BAL eosinophils from OVA-sensitized and -challenged hFcεRIα mice, hFcεRI was cross-linked with either cIgE Ab and mouse anti-human IgE (left panel) or anti-hFcεRIα mAb (15.1) and anti-mouse IgG1 (right panel). Histograms show expressions of costimulatory proteins on resting eosinophils (middle histograms) and FcεRI cross-linked eosinophils (bold line right histograms) vs isotype control Ab (left histograms). (**D**) Western blotting shows that hFcεRIα cross-linking, elicited by 15.1 mAb + anti-mIgG1 and cIgE^+^ anti-hIgE, increases CD40, CD80, and CD86 proteins in eosinophils. Eosinophils were treated with PBS, control mIgG1 mAb, 15.1 mAb only, 15.1 mAb + anti-mIgG, or cIgE only, cIgE + mouse isotype IgG, and cIgE+ anti-hIgE.

We investigated whether IgE/FcεRI-facilitated Ag uptake by airway eosinophils enhances presentation of Ag to Ag-specific T cells in pLNs. A nitrophenyl (NP)-specific chimeric human IgE (cIgE, bearing human IgE Fc) Ab allows the targeting of NP-OVA ex vivo in a fashion similar to that happening in allergic lungs where hFcεRI^+^ eosinophils encounter allergens in the presence of allergen-specific IgE. FcεRIα^+^ eosinophils, pretreated ex vivo with NP-OVA and anti-NP cIgE complexes and instilled i.t., presented NP antigen to pLN T cells much more effectively than those pretreated with NP-OVA only or NP-OVA with non-specific human IgE, as assessed by pLN T cell proliferation (Figure 2A). This increased IgE-dependent eosinophil APC function was fully inhibited by a human α chain specific, blocking anti-hFcεRIα (15.1) mAb in a dose-dependent manner (Figure 2A), confirming that the humanized FcεRIα receptor on eosinophils mediated this IgE-facilitated Ag presentation.

### 3.5. IgE/FcεRI-Facilitated Eosinophil Antigen Presentation Enhances pLN IL-4 Secretion

Inhaled allergens may induce type 2 T cell-driven responses (Th2). We evaluated whether IgE/FcεRI facilitated eosinophil Ag presentation in vivo biases towards Th2 cytokine production by the pLN cells. Peritoneal eosinophils from hFcεRIα mice, pre-incubated ex vivo with NP-OVA or NP-OVA plus cIgE anti-NP, were introduced into the tracheas of NP-OVA-immunized mice. Three days later, increased pLN IL-4 production was elicited by eosinophils pre-incubated with NP-OVA and cIgE anti-NP, but not with control non-specific human IgE (Figure 2B). The IgE/FcεRIα-mediated IL-4 increase was completely inhibited by the addition of anti-FcεRIα mAb (15.1) in the pre-incubation period of eosinophils and NP-OVA-cIgE anti-NP complexes (Figure 2B). In contrast, INF-γ production by pLN cells was not altered (Figure 2B), indicating that IgE-FcεRI facilitated eosinophil antigen presentation may be biased towards Th2 cytokine production.

### 3.6. hFcεRI Cross-Linking Increases Eosinophil Expression of Co-Stimulatory Proteins

Activation of T lymphocytes requires signals not only from interactions of T-cell receptors with MHC/peptide complex on APCs but also from co-stimulatory stimuli. We investigated the effects of cross-linking hFcεRI on the expressions of co-stimulatory molecules CD40, CD80, and CD86 by airway eosinophils from BAL of OVA-challenged hFcεRIα mice. By flow cytometry, cross-linking of hFcεRI both with cIgE plus anti-human IgE and with anti-hFcεRIα mAb (15.1) plus anti-mIgG1 Ab increased CD40, CD80, and CD86 surface expressions on airway BAL eosinophils (Figure 2C). Cross-linking hFcεRIα also increased total amounts of CD40, CD80, and CD86 proteins in eosinophils (Figure 2D), confirming that cross-linking of hFcεRIα on eosinophils elicits enhanced eosinophil expressions of CD40, CD80, and CD86 proteins.

## 4. Discussion

We evaluated potential APC functions for eosinophils that may be mediated through IgE and the high-affinity IgE receptor, FcεRI, on eosinophils. Human eosinophils expressing FcεRI have been recognized in sites of allergic and immune responses, including in allergen-induced late-phase cutaneous reactions [16], in the BAL of allergen-challenged atopic asthmatics [18], in allergen-induced rhinitis [17], and in bullous pemphigoid [24]. To date, however, considerable uncertainties remain about the function of FcεRI on human eosinophils. Unlike acute “effector” responses of mast cells and basophils triggered through IgE and their tetrameric (αβγ_2_) chain complex FcεRIs, human eosinophils potentially activated through any IgE receptors failed to secrete cationic granule proteins (eosinophil-derived neurotoxin or eosinophil peroxidase) [2,25,26]. Moreover, IgE failed to elicit generation of superoxide anion or enhanced leukotriene C_4_ formation by human eosinophils [2,26]. Contributing further to uncertainties about FcεRI’s effects on human eosinophils, there have been studies of human eosinophil–FcεRI expression that, by flow cytometry, either failed to detect or found only low levels of FcεRI on blood eosinophils with no or marginal correlations between serum IgE levels and eosinophil surface FcεRI expression [27,28].

Nevertheless, in the face of these uncertainties of eosinophil FcεRI surface expression and function, human eosinophils intracellularly contain substantial preformed pools of FcεRI α chain protein. Using human α chain-specific anti-hFcεRIα (15.1) mAb (as we used) to detect α chain, eosinophils had minimal (~4500) mAb binding sites on their surfaces yet contained 50,000 mAb binding sites intracellularly [6]. Seminaro et al., with other FcεRI Abs, found that human eosinophils, despite their undetectable hFcεRI cell surface expression, nevertheless contained substantial intracellular pools of hFcεRIα chain protein [27]. A subsequent study, while confirming that blood eosinophils from atopic donors expressed negligible surface expressions of FcεRI, also found that human eosinophils, by flow cytometry and immunostaining, contained intracellular FcεRIα chain protein as well as mRNAs for α and γ chains of the FcεRI [29]. An analysis based on next-generation RNA sequencing demonstrated that human eosinophils from normal donors and eosinophil-associated diseases expressed mRNAs for the α and γ chains of the trimeric eosinophil FcεRI receptor, albeit at low levels [3].

While studies have failed to find roles for FcεRI in mediating conventional eosinophil “effector” responses, eosinophils are recognized for other, often immunomodulatory, roles, including serving as APCs [30]. Studies with other cells, including dendritic and Langerhans cells, documented roles of hFcεRIα in mediating APC functions of these cells [5,31,32]. Our prior work established eosinophils as “professional” APCs able to present Ag to Ag-primed and naïve T cells [9,10]. In the present study we investigated the role of FcεRI in enhancing eosinophil APC function. Both peritoneal and endobronchial eosinophils from hFcεRIα transgenic mice express the humanized FcεRI with higher levels of BAL eosinophils from sensitized and aerosol Ag-challenged mice (Figure 1A). In OVA-sensitized and -challenged mice, airways eosinophils from hFcεRIα mice internalized more Texas Red-OVA than control WT BALB/c mice that lack an IgE receptor did (Figure 1B) [20]. The humanized FcεRI from hFcεRIα mice recognizes both human and murine IgE [33]. Similar to IgE accumulations in airway secretions in human asthmatics, elevated levels of free mouse IgE are present in airway secretions of OVA-sensitized and -challenged mice [34]. Therefore, OVA Ag-specific mouse IgE in airway secretions may form Ag–IgE complexes that are internalized by airway eosinophils via FcεRI. This may explain why FcεRI^+^ airway eosinophils in OVA-sensitized and -challenged hFcεRI mice took up more i.t. instilled Texas Red-OVA, compared with FcεRI^-^ airway eosinophils in OVA-sensitized and -challenged WT mice (Figure 1B).

Airway eosinophils can migrate from the airways into regional lymph nodes [9,10]. In this mouse model of allergic airways inflammation, luminal eosinophils also trafficked more distantly into the spleen (Figure 1C,D). Migrating eosinophils were sensitively detected with a dual-labeling strategy employing hFcεRIα mouse eosinophils (detectable with FITC-labeled anti-hFcεRIα-specific 15.1 mAb), previously labeled ex vivo with a red fluorescent dye DiIC_16_. Splenic, but non-PLN, sequestration of eosinophils was observed when eosinophils were intravenously injected into recipient mice, consistent with prior findings [12]. Eosinophil trafficking from the airway lumen into the spleen provides a possibility for Ags inhaled into the airways to be processed and transported into lymphoid organs more distantly than regional lymph nodes to stimulate T cell responses.

To address the functionality of human eosinophil hFcεRIα, we investigated whether IgE-FcεRI-facilitated Ag uptake by airway eosinophils enhances presentation of Ag to Ag-specific T cells in pLNs in vivo. A nitrophenyl (NP)-specific chimeric human IgE (cIgE, bearing human IgE Fc) Ab allows the targeting of NP-OVA ex vivo in a fashion similar to that happening in allergic lungs where hFcεRI^+^ eosinophils encounter allergens in the presence of allergen-specific IgE. FcεRIα^+^ eosinophils, pretreated ex vivo with NP-OVA and anti-NP cIgE complexes and instilled i.t., presented NP antigen to pLN T cells much more effectively than those pretreated with NP-OVA only or NP-OVA with non-specific human IgE, as assessed by pLN T cell proliferation (Figure 2A). In addition, this increased eosinophil APC function was inhibited by a human α chain-specific and blocking anti-hFcεRIα (15.1) mAb in a dose-dependent manner (Figure 2A), confirming that the humanized FcεRIα receptor on eosinophils mediated this IgE-facilitated Ag presentation.

In addition to demonstrating Ag-specific IgE-hFcεRIα-meditated pLN cellular proliferation, pLN cells increased IL-4 secretion in response specifically to NP-OVA and anti-NP cIgE that was inhibited by blocking IgE-hFcεRIα with α chain-specific 15.1 mAb. (Figure 1B) PLN cells would include resident cells as well as recruited eosinophils so the cellular source(s) of IL-4 (vs IFN-γ) were not defined but reflect a Th2 bias.

In support of roles of IgE-hFcεRIα-mediated eosinophil APC function, FcεRI cross-linking increased CD40, CD80, and CD86 expressions on hFcεRIα eosinophils. Engaging and cross-linking IgE-hFcεRIα by two approaches (cIgE or 15.1 mAb) both enhanced eosinophil cell surface expressions of CD40, CD80, and CD86 (Figure 2C). Western blotting confirmed the enhanced eosinophil contents of CD40, CD80, and CD86 (Figure 2D). Our findings showed that IgE-hFcεRI-dependent upregulation of co-stimulatory CD80, CD86, and CD40 on hFcεRI eosinophils would be consonant with a recent study of 32 allergic asthma subjects who had clinically beneficial responses over 16 weeks of treatment with the anti-IgE mAb, omalizumab [35]. As assessed on blood eosinophils by flow cytometry, omalizumab responders had significantly diminished expressions of CD40, CD80, and CD86 on eosinophils [35]. Those findings indicating that IgE can augment on human eosinophils the eosinophils’ lymphocyte co-stimulatory proteins would be in line with our findings that activation of hFcεRI by cross-linked IgE and by cross-linked α chain specific 15.1 mAb both enhanced CD40, CD80, and CD86 expressions on eosinophils (Figure 2C,D).

Our studies documented a role of IgE-hFcεRIα in mediating IgE-directed eosinophil APC function, distinct from the classic “effector” responses of eosinophils. Moreover, we addressed some of the uncertainties that have questioned the functional roles of FcεRI on eosinophils. The eosinophil hFcεRI, as found on other APCs, is a trimeric single α chain and dual gamma chain (αγ_2_) receptor lacking a beta chain as expressed on mast cells and basophils. Enhanced surface expression of FcεRI on mast cells and basophils is attributable to beta chain stabilization of surface-expressed receptors. Since eosinophil FcεRI lacks a beta chain, enhanced surface expressions of FcεRI on eosinophils may not be expected and are not usually found. Our findings also addressed the likely roles of intracellular α chain since its role in the trimeric eosinophil IgE-hFcεRIα is mediated by its intracellular pool that is endocytosed and is not dependent on new mRNA transcripts [8]. Hence, low levels of mRNA [3] are not informative measures of the functions of the α chain in eosinophil FcεRI. In summary, using humanized hFcεRIα expressed on mouse eosinophils, a functional, non-“effector” role for FcεRI on eosinophils is demonstrated identifying a means by which IgE may act on eosinophils to mediate their immunomodulatory, enhanced antigen presentation capabilities.

## Data Availability

Data are available from the corresponding author.

## Supplementary Materials

The following supporting information can be downloaded at: https://www.mdpi.com/article/10.3390/cells14040301/s1, Figure S1. (A) No hFcεRIα (vs isotype control mAb) is detected on polymyxin-elicited peritoneal eosinophils and on BAL eosinophils of WT BALB/c mice. (B) The mean fluorescent intensity (MFI) obtained by flow cytometry indicates the expression levels of hFcεRIα on polymyxin-elicited peritoneal eosinophils and BAL eosinophils from hFcεRIα transgenic mice. Data represent means ± SD of three mice. * *p* < 0.01 indicates statistical significance when comparing the BAL eosinophils with peritoneal eosinophils.

## Abbreviations

Ab	antibody
Ag	antigen
APC	antigen-presenting cell
BAL	bronchoalveolar lavage
cIgE	chimeric human IgE
DiIC_16_(3)	(1,1′-dihexadecyl-3,3,3′,3′-tetramethylindocarbocyanin perchlorate
FACS	fluorescent activated cytometer
FCS	fetal calf serum
hFcεRIα	humanized FcεRI α chain
i.t.	intratracheal
i.p.	intraperitoneal
LN	lymph node
mAb	monoclonal antibody
NP	4-Hydroxy-3-nitrophenyl acetyl
NP-OVA	4-Hydroxy-3-nitrophenyl acetyl (NP) coupled ovalbumin
OVA	ovalbumin
pLN	paratracheal lymph nodes
PBS	phosphate buffered saline
TBS	Tris buffered saline
WT	wild type
