# Supplementary material for: Humanized FcεRI Expressed on Mouse Eosinophils Mediates IgE-Facilitated Eosinophil Antigen Presentation
Source: Cells. 2025 Feb 18;14(4):301. doi: 10.3390/cells14040301 (PMC11854174; doi:10.3390/cells14040301)
Supplement: Supplementary file 1 [file cells-14-00301-s001.zip › cells-3451022-supplementary.pdf]

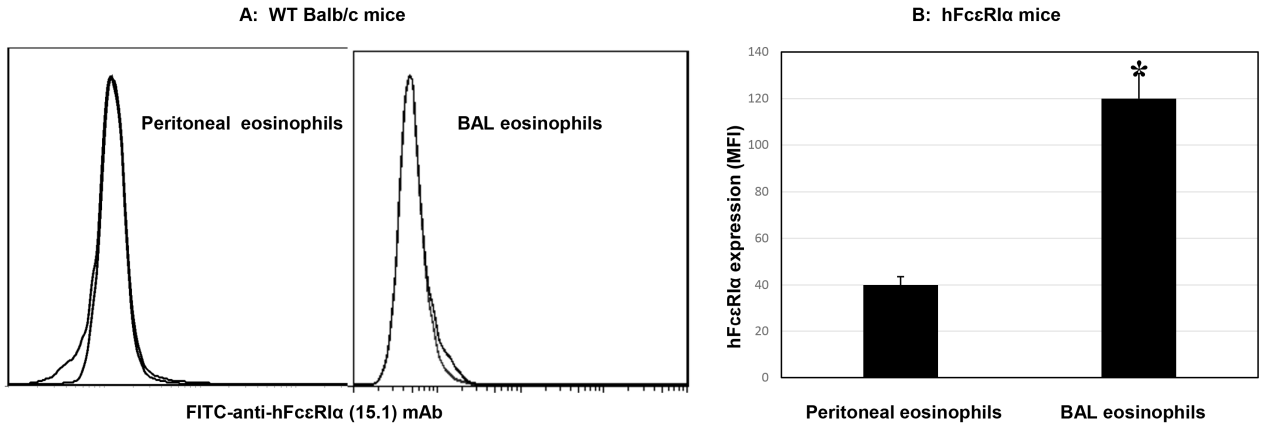

Figure S1. (A) No hFcεRIα (vs isotype control mAb) is detected on polymyxin-elicited peritoneal eosinophils and on BAL eosinophils of WT BALB/c mice. **(B)** The mean fluorescent intensity (MFI) obtained by flow cytometry indicates the expression levels of hFcεRIα on polymyxin-elicited peritoneal eosinophils and BAL eosinophils from hFcεRIα transgenic mice. Data represent means ± SD of three mice. *$p<0.01$ indicates statistical significance when comparing the BAL eosinophils with peritoneal eosinophils.
